# High-Frequency Cognitive Control Training for Depression: Case Report

**DOI:** 10.2196/56598

**Published:** 2024-11-29

**Authors:** Yannick Vander Zwalmen, Kristof Hoorelbeke, David Demeester, Ernst H W Koster

**Affiliations:** 1 Department of Experimental Clinical and Health Psychology Ghent University Ghent Belgium; 2 Department of Head and Skin Ghent University Hospital Ghent Belgium

**Keywords:** cognitive control training, CCT, cognitive function, depression, recurrence, relapse, prevention, case report, working memory, memory training, task performance, digital health

## Abstract

**Background:**

Cognitive control training (CCT) has gained attention in recent years as a preventative intervention in the context of major depressive disorder. To date, uncertainty exists around the working mechanisms of CCT and how its effects unfold overtime.

**Objective:**

This study aimed to examine cognitive and affective transfer effects following an unusually high number of training sessions.

**Methods:**

This case report presents data of a participant completing a large amount of training sessions (n=55) over the course of 1 year in 2 training phases: 10 initial sessions, followed by 45 additional sessions. Reliable change indices were calculated for several self-report questionnaires, measuring cognitive and affective functioning.

**Results:**

Cognitive task performance suggests improved cognitive functioning after training (accuracy scores increased from 43/181, 24% at baseline to 110/181, 61% shortly after training), which was maintained at follow-up (accuracy scores around 50%). Reliable change indices suggest a decrease in depressive symptoms (Beck Depression Inventory-II score decreased from 23 at baseline to 3 following initial training). Similarly, burnout symptoms following CCT showed a similar decrease. Maladaptive emotion regulation strategies displayed high variability, decreasing after periods of training but increasing when no training was performed. However, no changes in repetitive negative thinking were observed. Thematic analysis from an in-depth interview focusing on CCT adherence and user experience pointed to the importance of independency and accessibility of CCT in perceived agency, as well as the need for clear feedback mechanisms following training.

**Conclusions:**

Training task performance indicates further increases in performance beyond typical amounts of training sessions (10-20 sessions), hinting that more sessions could be beneficial for continued improvement in cognitive functioning. In line with previous research, CCT decreased depressive symptomatology. However, its effects on emotion regulation remain unclear. Further mechanistic studies into the temporal unfolding of CCT effects are necessary to investigate potential working mechanisms.

**Trial Registration:**

ClinicalTrials.gov NCT05166798; https://clinicaltrials.gov/study/NCT05166798

## Introduction

Cognitive impairment is one of the main symptoms of major depressive disorder and has repeatedly been shown to worsen after recurrent episodes [1,2]. In an effort to remediate these cognitive deficits, recent years have seen an increase in the use of cognitive control training (CCT) in the context of depression [3,4]. Systematic reviews and meta-analyses of CCT have reported significant small to medium effects on depressive symptomatology [5-7], and some clinical studies have found beneficial long-term effects of CCT for depression or relapse prevention of depression [3,8]. However, the working mechanisms of CCT are currently unclear (for a review, see von Bastian et al [9]). It is assumed that CCT results in observable transfer effects, such as task-specific transfer (ie, improvements on tasks very similar to the training), near transfer (ie, improvements in the trained cognitive construct, measured by a novel, training-unrelated task), and far transfer (ie, other cognitive and emotional constructs). Some empirical support has been found for mechanisms of task-specific and near transfer, such as the adoption of task-specific strategies [10,11] and improved attention allocation [12,13], but these alone are insufficient to explain far transfer. Cognitive theories often stress the importance of attentional or behavioral processes in repetitive negative thinking (RNT), and rumination in particular, which are known to be important risk factors for depression [14,15]. Indeed, these adaptive or maladaptive emotion regulation processes are known to be affected by cognitive control impairments and depression [16]. For instance, Ehring et al [17] found that individuals with remitted depression (RMD) reported greater difficulty in regulating negative emotions and more frequently used rumination when compared with a sample of participants who were never depressed. However, these groups did not differ on positive reappraisal and refocusing, suggesting that difficulties in reappraisal are more likely to be observed in samples with acute depression. Similarly, Hoorelbeke and Koster [18] found a significant decrease in the self-reported use of maladaptive strategies following CCT when compared with an active control condition in a sample with RMD.

Aside from these beneficial effects on maladaptive emotion regulation, multiple studies have reported positive effects of CCT on adaptive emotion regulation strategies. Adaptive emotion regulation strategies have also been found to be an important predictor of resilience, which allows maintaining one’s mental health even in confrontation with adversity [19]. For example, CCT has been linked to improvements in reappraisal [20] and positive emotion regulation [21]. Another recent study examined a dosage effect and found that nonemotional training reduced individuals’ emotional responses marginally after 7 days and significantly after 15-day training [22]. Despite these positive findings, it currently remains unclear how CCT results in these cognitive and emotional improvements, which necessitates more studies into the temporal unfolding of CCT effects.

CCT is often administered through adaptive cognitive tasks. One frequently used operationalization of CCT is the Adaptive Paced Auditory Serial Addition Task (aPASAT; [23]), which is an arithmetic task during which participants are presented with a stream of auditory digits (0-9) and are asked to calculate the sum of the last 2 heard digits. The aPASAT tailors its difficulty to individual performance levels: after 4 consecutive correct responses, the training speeds up by 100 ms, and after 4 consecutive incorrect responses, the training slows down by 100 ms. This adaptive nature of the training ensures that individuals need to focus their attention on the task despite making many errors, providing an intensive training experience. Recent meta-analytical research suggests that aPASAT training can be useful in the context of depression [7]. However, to identify how CCT could benefit individuals with depression or RMD, more mechanistic studies are required.

In this single-case report, we provide a detailed examination of change in cognitive task performance and self-reported well-being of an individual with RMD who performed a large amount of aPASAT training sessions (n=55). After a baseline assessment, the participant completed a first training phase consisting of 10 sessions (ie, the most widely used dosage for this type of training; [7]), which was followed by an extensive follow-up period, which included a second, elaborate training phase and final follow-up assessment. The training scheme used and high number of training sessions completed offer a unique opportunity to broadly map aPASAT training effects, where we closely monitored (1) cognitive functioning, using behavioral measures for task-specific and near cognitive transfer, and a self-report measure for effortful control; (2) severity of depressive symptomatology and burnout complaints; and (3) change in emotion regulation processes, focusing on RNT in particular, and general use of adaptive or maladaptive emotion regulation strategies. In addition, a structured interview was held to gather qualitative information about past and present depressive complaints, as well as to examine user experience of aPASAT training.

## Methods

### Overview

This paper reports the findings of a single case report, following an individual with RMD after inclusion in a preregistered randomized controlled trial (RCT; ClinicalTrials.gov identifier: NCT05166798; [24]). The RCT comprised of (1) a screening phase, (2) baseline assessment, (3) CCT, (4) posttraining assessment after four weeks, (5) three-month follow-up assessment, and (6) six-month follow-up assessment. This single-case report extended this design, including (7) an optional second wave of CCT and (8) a twelve-month follow-up assessment. The final assessment also included a qualitative interview focusing on training adherence, user experience, and treatment mechanisms. Phases 5, 6, and 8 are further referred to as 3-month follow-up, 6-month follow-up, and 12-month follow-up, respectively. This single-case report follows the Single-Case Reporting Guideline In Behavioural Interventions (SCRIBE; [25]), and all analyses can be reproduced from the data and analysis script in R (R Core Team), publicly stored on Open Science Framework [26].

The screening phase consisted of a telephone interview to determine if the participant met inclusion criteria, which were having a history of ≥ 1 depressive episode or episodes, being currently in remission (≥3 months), and having access to a computer with an internet connection. Exclusion criteria were having an ongoing depressive episode, previous or current psychotic disorder, neurological impairments, or excessive substance use. The use of antidepressant medication was allowed if kept at a constant level. Baseline and posttraining assessment dates were set, which took place at Ghent University. At baseline, the participant was briefed about the full study protocol and provided informed consent. The participant completed self-report questionnaires and 2 cognitive tasks (refer to Table 1 for an overview). The day after baseline, the participant started CCT on the online Cogtraining platform ([27]; for detailed information on the gamified training procedure and inclusion of individuals with RMD during the development of the platform, we refer to Vervaeke et al [28,29]) and completed 10 sessions. Four weeks after baseline, the participant was invited back for posttraining assessment, during which the same questionnaires and cognitive tasks as during baseline were administered. At 3 and 6 months after baseline, the participant received an invitation through email to fill in the self-report questionnaires and completed the PASAT at home. After completion of the 6-month follow-up, the participant received monetary compensation (€40 [US $43.17]) and a new account on the Cogtraining platform for optional further training. Although no further instructions were given after the completion of the initial study, several participants opted to continue their training freely. Among these, 2 participants performed more than 50 sessions. We invited these 2 participants for an additional follow-up to examine training progression, as well as the effects of an unusually high amount of aPASAT training sessions. A 57-year-old white male participant who completed 57 training sessions did not respond to the invitation. A second participant who completed a total of 55 sessions accepted our invitation and was invited back to Ghent University for an additional follow-up assessment and interview, which took place 12 months after baseline. Thematic analysis was used to detect and review important themes and was reported using data extracts, in line with recommendations by Braun and Clarke [30]. The participant was a 62-year-old white female individual with a master’s degree. She was married and, at baseline, was not working for 3 months due to a physical injury. Her first depressive episode occurred when she was 30 years old, and she reported 5 depressive episodes in her lifetime, reflecting a high risk for recurrence of depression. She reported a history of antidepressant medication use and psychotherapy but did not receive inpatient treatment for depression. At baseline, she no longer used antidepressant medication nor followed psychotherapy. Table 1 contains the self-report questionnaire and cognitive task performance scores from the longitudinal, single-case report examining the effects of high-frequency aPASAT training in an individual with RMD.

**Table 1:** Self-report questionnaires and cognitive task performance over time.

			Baseline assessment		Posttraining assessment		3-Month follow-up		6-Month follow-up		12-Month follow-up
**Subjective outcomes**											
	ATQ-EC^a^	3.7		3.9		3.7		3.5		4.3	
	BDI-II-NL^b^	23		3		19		7		9	
	RDQ^c^	62		5		40		6		10	
	BAT^d^	3.4		2.7		2.5		3		1.7	
	PTQ^e^	36		45		45		44		45	
	CERQ^f^ Adaptive	64		61		70		69		69	
	CERQ Maladaptive	27		20		26		30		24	
	CEQ^g^ Credibility	22		19		—^h^		—		19	
	CEQ Expectancy	11		23		—		—		15	
	LTE^i^	—		—		0		0		0	
**Objective outcomes**											
	PASAT^j^ (n=181), n (%)	43 (24)		110 (61)		85 (47)		96 (53)		112 (62)	
	Dual n-back	2.7		3.4		—		—		3.4	

^a^ATQ-EC: Adult Temperament Questionnaire, Effortful Control subscale.

^b^BDI-II-NL: Beck Depression Inventory, second edition, Dutch version.

^c^RDQ: Remission from Depression Questionnaire.

^d^BAT: Burnout Assessment Tool.

^e^PTQ: Perseverative Thinking Questionnaire.

^f^CERQ: Cognitive Emotion Regulation Questionnaire.

^g^CEQ: Credibility and Expectancy Questionnaire.

^h^Not applicable.

^i^LTE: List of Threatening Experiences.

^j^PASAT: Paced Auditory Serial Addition Task.

### Outcome Measures

Outcome measures consisted of self-report questionnaires and cognitive tasks. We included 3 measures of cognitive transfer. The task-specific transfer was assessed with a standardized version of the PASAT, using 3 blocks of 60 trials each, where the interstimulus interval (ISI) increased with each block, using an ISI of 3000 ms, 2000 ms, and 1500 ms for each block, respectively. Task-specific transfer was assessed using the mean accuracy over the 3 blocks. Near transfer was measured with a dual n-back task [31], which presented a visual (blue square) and auditory stimulus (letter) simultaneously. In total, 3 levels were used (1-back, 2-back, and 3-back), with 3 blocks consisting of 20 + n trials at each level. The measure of interest was the dependent variable as defined by Jaeggi et al [32], that is, the proportion of hits minus false alarms averaged over all n-back levels. In addition, we included an indicator for subjective cognitive functioning, for which we relied on the Effortful Control subscale of the Adult Temperament Questionnaire (ATQ-EC; [33,34]).

Depressive symptomatology was assessed with the Beck Depression Inventory, second edition, Dutch version (BDI-II-NL; range: 0-63; [35,36]) and the Remission from Depression Questionnaire (RDQ; range 0-82; [37,38]). For the RDQ, the total score was obtained by summing up item scores after reverse scoring items indicative of psychopathology, with lower total scores reflecting better functioning. The degree of burnout complaints was measured with the short version of the Burnout Assessment Tool (BAT; range 12-60; [39,40]). RNT was assessed with the Perseverative Thinking Questionnaire (PTQ; range 0-60; [41,42]). Maladaptive and adaptive emotion regulation strategies were measured with the Cognitive Emotion Regulation Questionnaire (CERQ; [43]). Subscales for adaptive strategies included acceptance, taking perspective, planning, positive reappraisal, and positive reflection. Maladaptive strategies assessed the use of catastrophizing, ruminating, blaming yourself, and blaming others. Compound scores were calculated (adaptive compound score: range 20-100, maladaptive compound score: range 16-80). The inclusion of the Credibility and Expectancy Questionnaire (CEQ; [44]) allowed us to investigate how the participant experienced the training procedure and whether her perspective on CCT changed over time. CEQ subscales were created in line with Devilly and Borkovec [44]: the first 3 items were used to create the credibility subscale and the last 3 items were used to create the expectancy subscale. Due to the design of the study, no *z* scores could be calculated, and thus, sum scores for each subscale were calculated. Finally, the possible influence of stressful life events was evaluated using the List of Threatening Experiences (LTE; [45,46]).

Data were analyzed by combining visual analysis [47] with calculated reliable change indices (RCIs). For the ATQ-EC, BDI-II-NL, RDQ, BAT, PTQ, and CERQ, RCIs were calculated by subtracting the previous score from the next test score, divided by the SE of the measure, which was calculated by multiplying the SD of the scale with the root of 1 minus the reliability of the scale [48,49]. RCIs were considered statistically significant if equal to or greater than 1.96, corresponding with =.05.



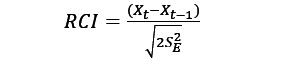





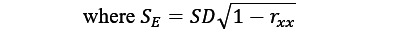



### Ethical Considerations

This research was reviewed and approved by the medical ethical committee of Ghent University Hospital (reference BC-10551). It was conducted in accordance with the 1964 Helsinki Declaration and its later amendments. The participant signed an informed consent form for both the initial as well as extended data collection. Publicly available data were anonymized for the protection of the privacy of the participants. No identification is possible in any of the images of the manuscript or Multimedia Appendix 1. The participant received a reimbursement of €90 (US $97.12).

## Results

### Training Performance

**Figure 1:**
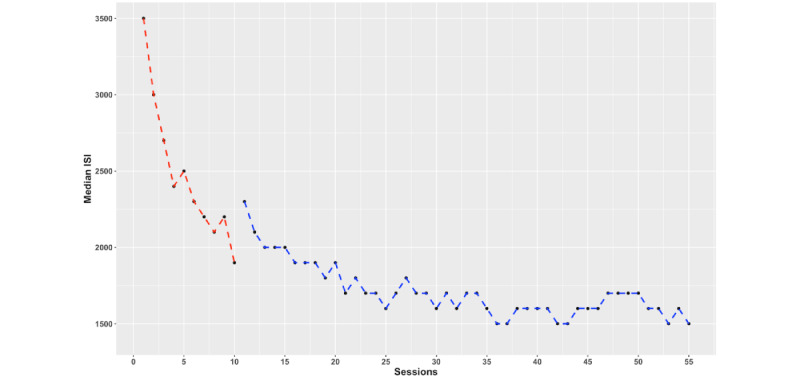
aPASAT training performance is measured using the median ISI. Training performance during the randomized controlled trial (10 sessions) is depicted with a red line. After 8.5 months, when the participant started freely training again, the median ISI values are shown in blue. Lower median ISI values indicate stronger cognitive performance. aPASAT: Adaptive Paced Auditory Serial Addition Task; ISI: interstimulus interval.

To examine training performance, the median ISI of each aPASAT training session was used, which typically presents as a logarithmic function, with early sessions seeing strong improvements in median ISI and evolving into a plateau. Figure 1 indicates that improvements in aPASAT performance continued during the initial training procedure, consisting of 10 sessions, and further improved during the second training phase, which the participant initiated 8.5 months following baseline. During the second training phase, the participant completed 45 training sessions over the course of 2.5 months (the last training session was completed 1 month before the final, 12-month follow-up). Interestingly, median ISI kept decreasing until around session 25, after which a plateau of around 1600 ms was obtained.

**Figure 2:**
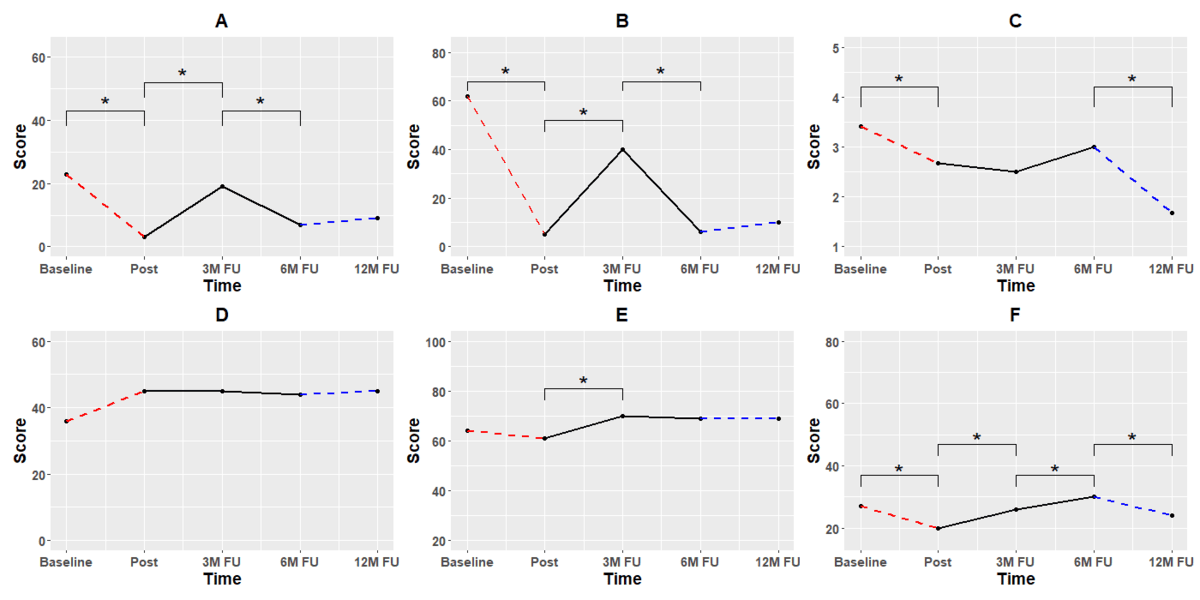
Subjective outcomes with y-scale from minimum to maximum of the outcome measure: (A) BDI-II, (B) RDQ, (C) BAT, (D) PTQ, (E) CERQ Adaptive subscales, and (F) CERQ Maladaptive subscales. Red dashed lines indicate that the first training period (10 sessions) took place between both assessments. Black lines indicate periods of no training. Blue dashed lines indicate that the second free training phase (additional 45 sessions) took place between both assessments. Asterisks indicate significant change according to RCIs. 3M: 3 months; 6M: 6 months; 12M: 12 months; BAT: Burnout Assessment Tool; BDI: Beck Depression Inventory; CERQ: Cognitive Emotion Regulation Questionnaire; FU: follow-up; PTQ: Perseverative Thinking Questionnaire; RCI: reliable change index. RDQ: Remission from Depression Questionnaire.

RCIs were calculated for the subjective outcome measures (refer to Table 2), which were then used in combination with visual analysis of the data to examine subjective differences over time. For an overview of the change in percentages of self-report questionnaires and cognitive task performance over time, please refer to Multimedia Appendix 1. Figure 2 provides an overview of clinically significant effects on self-report measures.

**Table 2:** Reliable change indices.

Outcome	Reliability of measure	SD of measure	Reference for reliability data	RCIs^a^ from baseline to posttraining assessment	RCIs from posttraining to 3-month follow-up	RCIs from 3- to 6-month follow-up	RCIs from 6- to 12-month follow-up
ATQ-EC^b^	α=.78	0.60	[50]	0.53	–0.53	–0.53	2.14^c^
BDI-II-NL^d^	α=.90	10.9	[51]	–4.10^c^	3.28^c^	–2.46^c^	0.41
PTQ^e^	*r*_tt_^f^=.69	9.99	[41]	0.84	0	–0.09	0.09
RDQ^g^	α=.85	16.4	[37]	–6.35^c^	3.90^c^	–3.79^c^	0.45
BAT^h^	α=.90	0.66	[40]	–2.54^c^	–0.58	1.69	–4.51^c^
CERQ^i^ Adaptive	α=.80	3.79	[43]	–1.2	3.76^c^	–0.42	0
CERQ Maladaptive	α=.80	2.95	[43]	–3.75^c^	3.22^c^	2.14^c^	–3.22^c^

^a^RCI: reliable change index.

^b^ATQ-EC: Adult Temperament Questionnaire, Effortful Control subscale.

^c^Equal to or greater than 1.96, indicating the reliable change.

^d^BDI-II-NL: Beck Depression Inventory, second edition, Dutch version.

^e^PTQ: Perseverative Thinking Questionnaire.

^f^Test-retest correlation.

^g^RDQ: Remission from Depression Questionnaire.

^h^BAT: Burnout Assessment Tool.

^i^CERQ: Cognitive Emotion Regulation Questionnaire.

### Cognitive Transfer Effects

Task-specific transfer was measured using accuracy scores on a nonadaptive PASAT. At baseline, the participant scored 43/181, 24%. After 10 aPASAT training sessions, she scored 61% (110/181), suggesting task-specific transfer. At 3 and 6 months, with no further training, scores lowered slightly to 47% (85/181) and 53% (96/181), respectively, suggesting maintained task-specific transfer. Finally, after 45 further training sessions, at the 12-month follow-up, she scored 62% (112/181), which is very similar to the posttraining measurement, suggesting that a ceiling effect was reached in terms of task-specific transfer.

**Figure 3:**
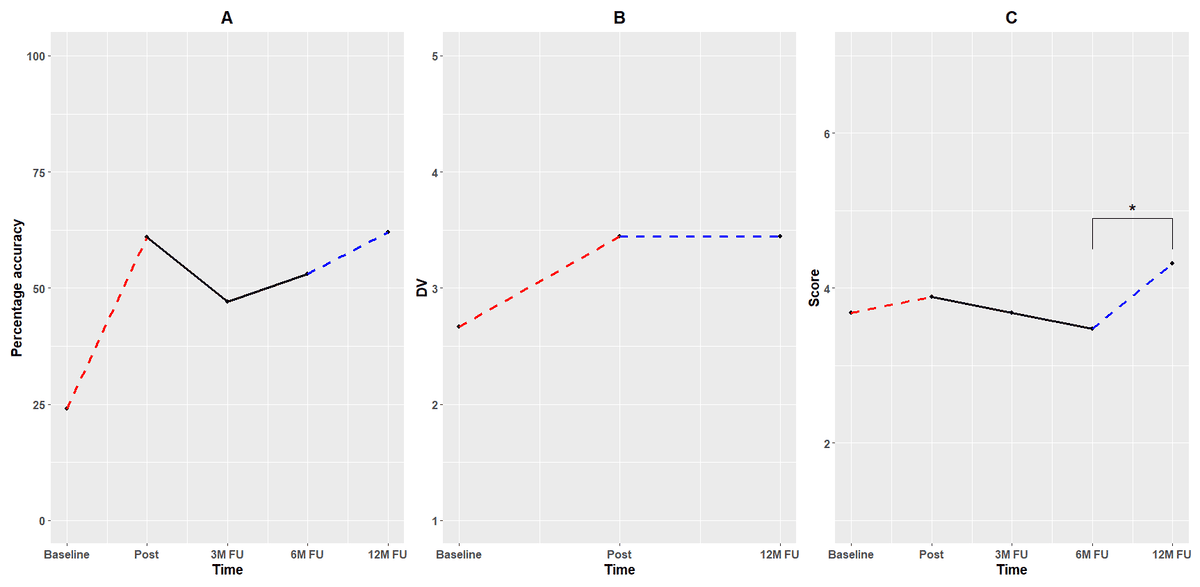
Task-specific and near transfer, with the (A) nonadaptive PASAT and (B) dual n-back, respectively, and (C) subjective effortful control (ATQ-EC). Red dashed lines indicate a period of training (10 sessions). Black lines indicate periods of no training. Blue dashed lines indicate further free training (additional 45 sessions). Asterisks indicate significant change according to RCI. RCI could only be calculated for the ATQ-EC; the absence of asterisks on PASAT and dual n-back is not indicative of the absence of effects on these measures. 3M: 3 months; 6M: 6 months; 12M: 12 months; ATQ-EC: Adult Temperament Questionnaire, Effortful Control subscale; FU: follow-up; PASAT: Paced Auditory Serial Addition Task; RCI: reliable change index.

To examine near transfer, a dual n-back was performed at baseline, posttraining measurement, and 12-month follow-up. It is interesting that near transfer effects, as measured by the dependent variable proposed by Jaeggi et al [31], were maintained from posttraining measurement to 12-month follow-up. Figure 3 shows both cognitive transfer tasks, as well as subjective effortful control (ATQ-EC).

Little variability was observed in subjective cognitive functioning as measured with the ATQ-EC, where only the 6- to 12-month follow-up saw a significant increase. This suggests that the initial 10 training sessions were not sufficient in increasing effortful control, but it is possible that the additional 45 training sessions contributed to an increase in effortful control.

### Emotional Transfer Effects

To visualize the data, subjective data were plotted with the minimum and maximum range as the y-axis limits. Visual inspection of the depressive symptomatology data suggests that the participant’s symptoms fluctuated over time. At baseline, she was indeed only partially recovered from depression (BDI=23). After 10 CCT sessions, at posttraining measurement, depressive symptoms decreased below subclinical levels (BDI=3). These depressive symptoms reappeared at 3-month follow-up (BDI=19) and subsided again at the 6-month (BDI=7) and 12-month (BDI=9) follow-ups. This suggests that 10 sessions of aPASAT training could have initially helped decrease depressive symptomatology but was not sufficient on its own to prevent the recurrence of depressive symptoms after 3 months. There was no meaningful difference between BDI scores at 6- and 12-month follow-ups. A very similar result followed from the RDQ data, also indicating a strong decrease from baseline to posttraining measurement, increased symptoms at 3-month follow-up, and then subclinical symptoms at 6- and 12-month follow-ups. The RCIs were also significant at all time points except between the last 2 follow-ups. In contrast, burnout complaints as assessed with the BAT show 2 significant decreases (from baseline to posttraining measurement and from 6- to 12-month follow-up), corresponding to the training periods.

Remarkably, the participant showed little variability in the PTQ, suggesting no clinically significant changes in RNT over time. This is interesting considering that cognitive functioning and depressive complaints do vary over time but are seemingly unrelated to the RNT in this participant. Visually, it seems that the use of emotion regulation strategies, as measured with the CERQ, changed little over time. However, relying on the RCIs, we observed several clinically meaningful differences. The intensity of maladaptive emotion regulation strategy use significantly changed over time and seemed to correspond with the observed patterns of effects on indicators of depressive symptomatology. That is, the observed decrease in the use of maladaptive emotion regulation strategies between baseline and posttraining measurement corresponds with the decrease in depressive symptoms and residual complaints from baseline to posttraining measurement. Next, an increase in the use of these maladaptive strategies aligns with an increase in depressive symptoms from posttraining measurement to 3-month follow-up. Interestingly, the use of maladaptive emotion regulation strategies further increased from 3- to 6-month follow-up, which was not reflected in depressive symptom levels during this period. Finally, a significant decrease in the use of maladaptive strategies was seen from 6- to 12-month follow-up, which corresponds with improvements in burnout complaints in the same period. It is clear that the use of maladaptive emotion regulation strategies fluctuated greatly. Interestingly, the decreases correspond with the periods when the participant was doing CCT and the increases align with periods during which no CCT took place.

For the compound measure of adaptive emotion regulation strategy use, we only observed a clinically significant increase between posttraining measurement and 3-month follow-ups. This suggests that, during the posttraining period (ie, between posttraining measurement and 3-month follow-up), the participant experienced a higher need for emotional regulation and was using these adaptive strategies more in the period preceding the first training phase. Importantly, increased use of adaptive emotion regulation strategies continued until 1-year follow-up.

### Credibility, Expectancy, and Life Events

**Figure 4:**
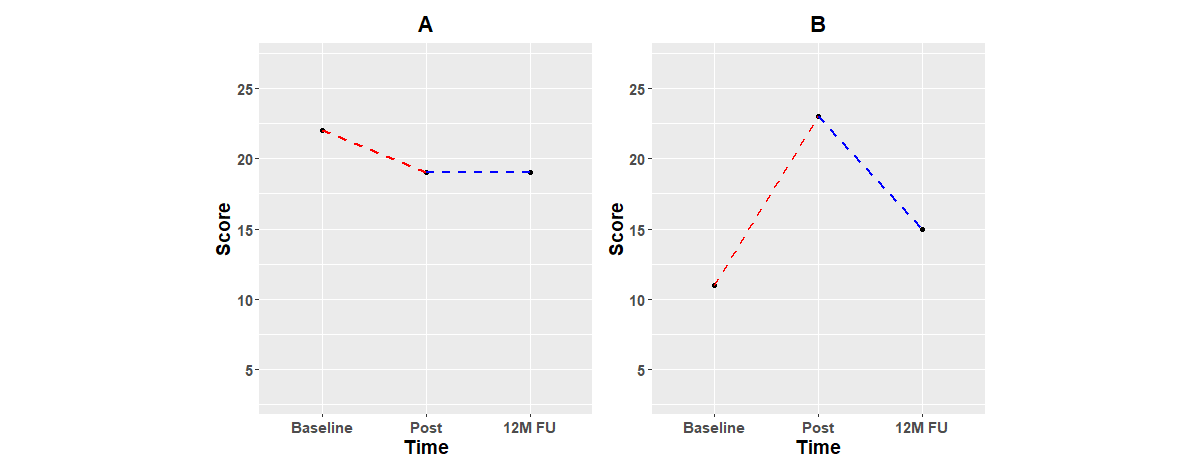
(A) Credibility and (B) expectancy as assessed with the CEQ. Red dashed lines correspond with the first training period (10 sessions). Blue dashed lines indicate that this period included the second free training phase (an additional 45 sessions). 12M: 12 months; CEQ: Credibility and Expectancy Questionnaire; FU: follow-up.

In total, 2 additional self-report questionnaires were used to check for potentially influencing variables, such as credibility and expectancy of the training (CEQ) and the potential occurrence of threatening life events (LTE). In the absence of RCI values for these measures, we strictly relied on visual interpretation. The credibility of the training (ie, how believable and logical CCT was), as measured with the CEQ, decreased slightly from baseline to posttraining measurement and remained constant at 12-month follow-up. Overall, credibility was relatively high, with scores ranging between 19 and 22 for all measurements (ie, possible values: 3-27), which is also reflected by the high amount of training sessions the participant completed. Indeed, if one did not find CCT logical for relapse prevention of depression, it would be unlikely to continue training to that extent. Expectancy (ie, how much the participant believed she would improve) saw a relatively large increase from baseline to posttraining measurement (from 11 to 23; possible values: 1-29), indicating that after the initial training, the participant expected her depressive symptoms to decrease. Compared with posttraining, lower expectancy scores were observed at 12-month follow-up (from 23 to 15), although these still appear to be slightly higher relative to baseline levels. At follow-up, the participant reported less depressive symptoms, which could indicate a lower need for additional training at that time and could help explain the slight decrease in expectancy. Both CEQ subscales are plotted with minimum and maximum values as the y-axis in Figure 4.

The participant did not report any important unpleasant or threatening occurrences for herself or loved ones, such as sickness, violence, relational or financial difficulties, at all 3 follow-up LTE questionnaires. In the “other” category, she reported feeling isolated due to her inability to work as a consequence of her physical injury at the first follow-up. In the first and last follow-up, she mentions that she worried about her adult child’s international career.

### Interview Analysis

Thematic analysis of the interview revealed three important themes: training experience, training duration, and training feedback.

### Training Experience

Throughout the interview, the participant mentioned the frustrating nature of the training and that she felt that she had a slow reaction speed:

I feel slow in my thinking and that of itself already gets on my nerves. When I do not feel well, I think I am probably even slower than usual.

Several times, she mentions thoughts about adaptive emotion regulation strategies, such as acceptance and positive reframing, which she used to decrease the frustration brought up by the training:

By doing the training more often, I have learned this is my capacity, I cannot go faster. Maybe other people are faster and better, but I will just do the best I can” and “I feel this training helped me get back into action, not to stay in the cocoon I had made. Overcoming these difficulties gives me satisfaction.

When asked what made her start doing CCT again between the 6- and 12-month follow-ups, she replied that she felt an increase in depressive symptoms and wanted to use something at her disposal to counter her perceived vulnerability. The participant stated that she liked that this CCT was something she could do herself when she was not feeling well, without having to rely on others (eg, clinicians). In particular, this seemed to fit with personal experiences related to the accessibility of health care services in rural regions.

I started doing the training again when I was starting to feel worse. If later in my life, I’m starting to feel indecisive again, I want to do the training again. I like that this training is something I can do myself, without having to go to other people. I would not be able to do this training if I needed to move to a clinical facility, that takes too much time and I live quite remote.

### Training Duration

Training duration was fixed at 15 minutes per session, which was considered to be an appropriate training duration. According to the participant, deviating from this may have resulted in poorer adherence. The participant expressed that how this period is perceived was contingent on her current level of functioning:

It felt as if the session [length] depended on how I felt that day. When I had a good day, it went by fast, but on a bad day, it went really slow. I have never not done the training because I thought it was too long, but it should not be longer than 15 minutes, because then I might not start the training.

When asked if she would like to be able to flexibly change the training duration for each session, depending on her current state of being, she said that she would not deem this feature to be helpful. In particular, the participant considered it useful to know that the duration of each training session was fixed, where she liked to be pushed a little:

No, I would not want to change the training duration. There are a lot of things in life you cannot escape, this is something you just have to learn, to keep going. This is the cognitive training, it is 15 minutes, you know this is what it is, so you just have to do it. Even when it is long, you have to learn to keep going in that moment.

### Training Feedback

Over the course of the interview, it became clear that some aspects of training feedback were not sufficiently clear. At the end of each session, participants are presented with graphs, detailing their accuracy and median speeds:

I found it hard to estimate how well I was doing the training. Some days I thought I did well, but it turned out I did not. I cannot estimate how well it went.

Part of the in-session feedback consists of a ringing tone after 4 consecutive correct answers, indicating an increase in speed by 100 ms. However, this was not clear to the participant:

Sometimes, a tone would ring, which was very distracting. I would prefer if that ringing tone was not present.

After informing the participant about the ringing tone logic, she said:

I never knew what that sound had to do with the training. I know that the training gets faster after 4 correct answers, but I never made the connection to the ringing sound.

## Discussion

### Principal Findings

CCT has been proposed as a possible intervention to remediate cognitive impairment in the context of depression [52]. In this case report, we studied the temporal unfolding of training effects over the course of an extended training period to generate new insights into the unfolding of transfer effects and to explore user experience in relation to extended training. For this purpose, we examined cognitive task performance and self-reported well-being of an individual with RMD who performed a large amount of aPASAT training sessions. Below, we first discuss the results of the self-report questionnaires, followed by cognitive task and training performance, and CCT adherence and experience.

The data suggest that CCT lowered depressive symptoms after initial training, but these effects did not last long term. Interestingly, after the second training phase (between 6- and 12-month follow-ups), no changes in depressive symptoms were observed. Qualitative analyses indicate that the participant restarted CCT during this period, as she was starting to experience more depressive symptoms again. During the interview, she mentioned that when she felt her depressive symptoms increase, she performed additional CCT sessions, which possibly helped her prevent a new depressive episode. This could explain the lack of change in depressive symptoms on the self-report measures between 6- and 12-month follow-ups. Burnout symptoms also decreased after the initial training phase, but remained constant afterward, until the second training phase, where we observed a further decrease in burnout symptoms. Together with the qualitative analyses, this aligns with the period in which the participant resumed work after her physical injury healed. Interestingly, burnout complaints were lower in a period when she was working, as opposed to a period when she was not working. Indeed, work-related stress factors are known to play a role in psychopathology, and the inability to work can lead to an increase in depressive symptoms [53].

Working mechanisms for CCT are currently not well understood (for review, see von Bastian et al [9]), but cognitive theories often highlight the importance of maladaptive emotion regulation, and RNT in particular [18]. Interestingly, despite reductions in depressive symptoms following CCT in this case report, RNT did not change over time. Several factors may have contributed to this unexpected pattern of findings. For instance, it is possible that CCT did not influence RNT processes in this participant, which would suggest that supposed working mechanisms do not always rely on RNT. Indeed, several factors can contribute to one’s pattern of RNT, of which cognitive control is only one [54]. Given the other effects on symptom level, this suggests that potentially other mechanisms or mediating factors could be at play. Previous studies examining the effects of CCT on rumination have indeed found inconsistent results, with some studies finding possible effects of CCT on rumination [55,56], whilst others found no such effects (eg, [29,57-59]). Heterogeneity between studies in terms of CCT operationalization, dose, sample population, etc, could play a role in these different findings. Another possible reason as to why the PTQ scores did not change over time could be because her worries did also not change over this time period. In the interview, as well as on the LTE questionnaire, at both baseline and follow-up measures, the participant mentioned being worried about the international career of one of her children.

Even though her level of RNT was constant, the use of maladaptive emotion regulation strategies varied across all timepoints. In addition to rumination, such strategies include self-blame, other-blame, and catastrophizing. Following both periods of training, she reported a decreased use of these maladaptive emotion regulation strategies. Interestingly, during periods of no training, these strategies were used more often. Indeed, cognitive control deficits have been associated with the use of maladaptive emotion regulation strategies [60], and cognitive control is required for the selection, monitoring, and flexible use of emotion regulation [61]. Interestingly, where CCT in this case report appeared to influence maladaptive emotion regulation strategies, an increase in cognitive control did not translate to a higher use of adaptive emotion regulation strategies. In the qualitative assessment, the participant frequently mentioned using several adaptive emotion regulation strategies such as reappraisal, acceptance, and putting things into perspective, but the self-report measures only found a small increase between posttraining measurement and 3-month follow-up. This pattern of findings could suggest that, in order for the effects of CCT on adaptive emotion regulation strategy use to develop, the use of maladaptive emotion regulation strategies first needs to decrease. Based on the current data and in line with existing literature (eg, [62]), deployment of maladaptive emotion regulation strategies seems more susceptible to CCT than adaptive emotion regulation use.

Related to the question of training dosage, aPASAT performance kept improving further than the typical number of sessions that participants have done in previous aPASAT RCTs (ie, mostly 10, maximum 12l refer to Vander Zwalmen et al [7]), suggesting that a higher number of sessions might continue to improve working memory. This case report found task-specific cognitive transfer, which is in line with most CCT research [9]. Although the data suggest an increase in dual n-back performance after initial training, which was maintained long term, there were insufficient measurements to convincingly show that near transfer occurred, since learning effects from baseline to posttraining measurement cannot be ruled out. Interestingly, for both cognitive tasks, posttraining and 12-month follow-up measures are strikingly similar, suggesting increased task performance but also a potential ceiling effect on these outcomes. Although training performance indicators kept improving beyond the posttraining measurement, the nonadaptive PASAT and dual n-back scores did not. It is currently unclear if continued training beyond the maximum task performance indicators would be beneficial.

During the qualitative assessment, the participant mentioned that she greatly values the accessibility of the training. Indeed, one of the core strengths of CCT, compared with other interventions in the context of depression, is its accessibility, for which only a computer with an internet connection is required. While other interventions often come with some form of waiting list or require additional steps (eg, transport to clinical practice), CCT can be done at home when it suits the individual, which was highly appreciated by the participant. Although it has been reported that some clinical guidance is preferable in CCT (eg, to avoid reinforcement of feelings of failure following stressful cognitive training; [28]), participants who have received psychoeducation can benefit from CCT in the form of a semiguided training.

In terms of training experience, a previous aPASAT user requirements analysis [28] found that response-related feedback both during and after training is important for treatment adherence, as well as improving training performance. Furthermore, previous studies using aPASAT training for depression used slightly different performance feedback mechanisms. Earlier studies typically kept performance feedback to a minimum [18,55], while more recent studies provided a more detailed form such as auditory [63] or visual feedback [64]. The aPASAT training that was used for this study was performed on the Cogtraining platform of Ghent University [27], which combined auditory and visual feedback. An auditory jingle was played after 4 consecutive correct answers (ie, when the training sped up with 100 ms), and visual feedback was displayed using green lights for correct answers and red lights for incorrect answers. During the structured interview, the participant mentioned that not all built-in feedback mechanisms worked well for her. The graphs after training and the in-session jingle indicating an increase in training speed were not always clear, suggesting that there is room for improvement in these feedback mechanisms.

This single-case report was the first to examine transfer effects following a high amount of aPASAT training sessions. Examination of training task performance suggested that training beyond typical doses (ie, circa 10 sessions) can yield additional performance gains, which could potentially lead to stronger effects. Future research should examine if this result could also be applicable to a broader population or if this was related to personal factors (ie, age, education level, and personality factors). More mechanistic studies are required to examine the working mechanisms behind CCT. For instance, single-case experimental designs could be used to frequently assess cognitive and emotional functioning before, during, and following CCT, allowing for a closer examination of temporal effects. Another important goal would be to investigate individual differences in treatment response and what factors influence its variability. If these findings can be reproduced in a larger sample, higher training doses or the use of booster sessions could be considered to improve the effects of CCT. However, such future research will have to examine if these higher training doses are feasible. While these may be doable for some individuals, they might not be attainable for everyone.

Several limitations of this case report should be considered. First, due to the use of offline measurement of the dual n-back, near transfer was only assessed at 3 time points, when the participant returned to the laboratory. As such, this did not allow us to sufficiently chart fluctuations over time. Second, due to the fact that no previous research has investigated the effects of aPASAT training using such a high training dosage, no comparison to other individuals was possible. This means that test-retest–type effects cannot be ruled out. Furthermore, the additional training sessions were performed by the participant of her own volition. This suggests that the training was both feasible and worthwhile but could also introduce bias. Third, the participant scored relatively high on credibility and expectancy, which could have contributed to possible placebo effects. Finally, although a single-case design is suited to examine temporal effects in 1 participant, these findings will have to be replicated in trials with a larger sample size to extrapolate these findings to the population with RMD.

### Conclusion

Overall, this case report provided evidence for improvements in depressive and burnout complaints following CCT, as well as continued training performance improvements beyond typical training doses. In addition, the use of maladaptive emotion regulation strategies fluctuated over time, when adaptive emotion regulation strategies and RNT did not. Qualitative data point to the importance of accessibility of CCT and suggest that future CCT implementations include a focus on clear training feedback features. More mechanistic research is needed to map mechanisms underlying effects of CCT so that improvements can be made to increase its efficacy and effectiveness.

## Appendix

Multimedia Appendix 1 Change in percentages for self-report questionnaires and cognitive task performance over time.

## Abbreviations

aPASAT: Adaptive Paced Auditory Serial Addition Task
ATQ-EC: Adult Temperament Questionnaire
BAT: Burnout Assessment Tool
BDI-II-NL: Beck Depression Inventory, second edition, Dutch version
CCT: cognitive control training
CEQ: Credibility and Expectancy Questionnaire
CERQ: Cognitive Emotion Regulation Questionnaire
ISI: interstimulus interval
LTE: List of Threatening Experiences
PASAT: Paced Auditory Serial Addition Task
PTQ: Perseverative Thinking Questionnaire
RCI: reliable change index
RCT: randomized controlled trial
RDQ: Remission from Depression Questionnaire
RMD: remitted depression
RNT: repetitive negative thinking
SCRIBE: Single-Case Reporting Guideline In Behavioural Interventions

## Data Availability

The datasets generated during and/or analyzed during this study are available in the Open Science Framework repository [26].
