# Supplementary material for: High-Frequency Cognitive Control Training for Depression: Case Report
Source: JMIR Form Res. 2024 Nov 29;8:e56598. doi: 10.2196/56598 (PMC11645507; doi:10.2196/56598)
Supplement: Multimedia Appendix 1 [file formative_v8i1e56598_app1.docx]

**Supplementary material**

Change in percentages for self-report questionnaires and cognitive task performance over time

|  | Baseline to Post | Post to 3 month follow-up | 3 month to 6 month follow-up | 6 month to 12 month follow-up |
| --- | --- | --- | --- | --- |
| *Subjective outcomes* |  |  |  |  |
| ATQ-EC | +5.4% | -5.1% | -5.4% | **+22.9%*** |
| BDI-II-NL | **-87.0%*** | **+533.3%*** | **-63.2%*** | +28.6% |
| RDQ | **-91.9%*** | **+700%*** | **-85.0%*** | +66.7% |
| BAT | **-20.6%*** | -7.4% | +20.0% | **-43.3%*** |
| PTQ | +25.0% | 0.0% | -2.2% | +2.3% |
| CERQ - adaptive | -4.7% | **+14.8%*** | -1.4% | 0.0% |
| CERQ - maladaptive | **-25.9%*** | **+30.0%*** | **+15.4%*** | **-20.0%*** |
| CEQ - credibility | -13.6% | n/a | n/a | n/a |
| CEQ - expectancy | +109.1% | n/a | n/a | n/a |
| LTE | n/a | n/a | 0.0% | 0.0% |
| *Objective outcomes* |  |  |  |  |
| PASAT (%) | +154.2% | -23.0% | +12.8% | +17% |
| Dual n-back | +25.9% | n/a | n/a | n/a |

*Note:* Where reliable change indices were significant, values are followed by an * and depicted in bold. *ATQ-EC* Adult Temperament Questionnaire, Effortful Control subscale, *BDI-II-NL* Beck Depression Inventory II, *BAT* Burnout Assessment Tool, *CERQ* Cognitive Emotion Regulation Questionnaire, *PTQ* Perseverative Thinking Questionnaire, *RDQ* Remission from Depression Questionnaire, *CEQ* Credibility and Expectancy Questionnaire, *LTE* List of Threatening Experiences, *PASAT* Paced Auditory Serial Addition Task, *n/a* not applicable
